# Morphological and Phenetic Evidence Support the Recognition of a New Limestone-Dwelling Species of *Boesenbergia* Kuntze (Zingiberaceae) from Northeastern Thailand †

**DOI:** 10.3390/life16081218

**Published:** 2026-07-23

**Authors:** Thawatphong Boonma, Surapon Saensouk, Piyaporn Saensouk, Charun Maknoi, Suriya Phimpha, Prasert Ruannakarn, Win Paing Oo

**Affiliations:** 1 Diversity of Family Zingiberaceae and Vascular Plant for Its Applications, Walai Rukhavej Botanical Research Institute, Mahasarakham University, Maha Sarakham 44150, Thailand; boonma.thawat@gmail.com; 2 Diversity of Family Zingiberaceae and Vascular Plant for Its Applications, Department of Biology, Faculty of Science, Mahasarakham University, Maha Sarakham 44150, Thailand; 3 Bureau of Research and Conservation, Queen Sirikit Botanic Garden, Botanical Garden Organization, Chiang Mai 50180, Thailand; charun@qsbg.org; 4 Faculty of Humanities and Social Sciences, Mahasarakham University, Maha Sarakham 44150, Thailand; suriya.pi@msu.ac.th; 5 Department of Educational Research and Development, Faculty of Education, Mahasarakham University, Maha Sarakham 44000, Thailand; prasert.rua@msu.ac.th; 6 College of Sustainability and Tourism, Ritsumeikan Asia Pacific University, 1-1 Jumonjibaru, Beppu 874-8577, Oita, Japan; winpaingoo2019@gmail.com

**Keywords:** *Boesenbergia*, limestone, new species, phenetic analysis, taxonomy, Thailand, Zingibereae

## Abstract

Limestone habitats in Southeast Asia often harbour narrowly distributed plant diversity, and several limestone-associated taxa of *Boesenbergia* require further taxonomic documentation. Field surveys and ex situ observations of living material from Loei Province, northeastern Thailand, revealed a distinctive *Boesenbergia* population associated with shaded limestone microhabitats. This study evaluates this population using comparative morphology, herbarium and literature comparisons, phenetic analyses based on 25 selected morphological characters, and a preliminary conservation assessment. *Boesenbergia lamudiae* Boonma, Saensouk & P.Saensouk, sp. nov., is described and illustrated. The species is morphologically most similar to *B. burmanica* Boonma, P.Saensouk & Saensouk, and resembles *B. rotunda* sensu lato in having a terminal inflorescence enclosed within the leaf sheaths and lacking an androecial cup. It differs by its broadly elliptic to ovate-elliptic laminae with an arachnoid abaxial surface, maroon lanceolate bracts, longer pubescent calyx with an acute to acuminate apex, non-saccate obovate labellum with a sharply delimited maroon transverse colour zone, larger anther crest, longer epigynous glands, and pubescent ovary. Principal coordinates analysis (PCoA) and unweighted pair group method with arithmetic mean (UPGMA) clustering based on Gower distance separated *B. lamudiae* from all compared operational units, supporting its morphological distinctiveness. The species is currently known only from Nong Hin District and is provisionally assessed as Critically Endangered (CR) under criterion D.

## 1. Introduction

The genus *Boesenbergia* Kuntze, belonging to the family Zingiberaceae and placed within the tribe Zingibereae, comprises 102 accepted species [1] distributed mainly throughout tropical Asia, from India across southern China and mainland Southeast Asia to the Malesian region, extending further south to the Lesser Sunda Islands and east to the Philippines [1,2,3,4,5,6,7,8,9]. Members of the genus are typically found in forest understorey habitats, although several species also occur in more specialized environments, including limestone areas, seasonal forest margins, and open plateaux [7,8,9,10,11]. Limestone habitats are of particular interest because they often form isolated ecological units with shallow soils, exposed rock surfaces, and heterogeneous shaded microhabitats. In Thailand, limestone areas have repeatedly yielded narrowly distributed and previously overlooked plant taxa, including several limestone-associated species of Zingiberaceae [12,13,14,15]. These findings indicate that limestone habitats may function as microhabitats for rare, localized, and endemic plant diversity, and emphasize the need for continued floristic and taxonomic surveys.

The circumscription of *Boesenbergia* has undergone considerable taxonomic revision over time, including the transfer of species from genera such as *Caulokaempferia* and *Haplochorema*, as well as the synonymization of *Jirawongsea* based on combined morphological and molecular evidence [16]. These changes have broadened the morphological limits of the genus, particularly with respect to floral structure, including variation in labellum shape, anther crest morphology, and the presence or absence of an androecial cup [16,17]. As a result, species delimitation within *Boesenbergia* remains challenging, especially in groups exhibiting overlapping vegetative characters or variable floral coloration.

One such problematic complex centres around *Boesenbergia rotunda* (L.) Mansf., a widely distributed and morphologically variable species [1,2,3,4,7,9,11]. Across its range, *B. rotunda* shows considerable variation in plant size, leaf morphology, indumentum, flower length, labellum shape, and floral coloration, particularly in the labellum, which may display diverse combinations of white, pink, red, or violet tones [2,4,9,11,17]. The colour pattern of the labellum is often diffuse, spotted, striped, or radiating, and the presence or intensity of markings may vary among regional descriptions. This high degree of variation has led to a broad species concept, under which several morphologically distinct taxa have been placed in synonymy.

However, recent re-examination of morphological characters, especially floral traits, suggests that this broad circumscription may obscure distinct species-level diversity. Characters such as labellum shape and colour pattern, indumentum of vegetative and floral parts, calyx length and apex morphology, ovary indumentum, and androecial features, including filament pigmentation and anther crest morphology, appear to be more stable and taxonomically informative than previously recognized [2,11]. In addition, the absence of an androecial cup defines a morphologically coherent group within *Boesenbergia*, within which several superficially similar species occur. Delimitation of species in this group requires careful evaluation of floral morphology based primarily on living material, because herbarium specimens alone may not adequately preserve critical characters such as colour pattern, floral orientation, labellum texture, indumentum, and fine structural details.

Living material of *Boesenbergia* cultivated at Brio Botanical Research Garden, Nakhon Nayok, Thailand, has flowered regularly since 2019, allowing for detailed observation of floral morphology over multiple seasons. Among these collections, plants derived from material originally collected from Nong Hin District, Loei Province, northeastern Thailand, were found to possess a distinctive and stable combination of morphological characters. The same diagnostic characters were also observed in the natural population again in 2025, indicating that they are not transient abnormalities or characters induced only under cultivation. The Loei material is morphologically most similar to *B. burmanica* Boonma, P.Saensouk & Saensouk and also resembles *B. rotunda* sensu lato in its terminal inflorescence enclosed within the leaf sheaths and absence of an androecial cup. Nevertheless, it differs from these and other related taxa by its unusually long pubescent calyx with an acute to acuminate apex, non-saccate obovate labellum with a sharply delimited deep reddish to maroon transverse band across the throat, larger recurved anther crest, and pubescent ovary.

This study hypothesizes that the limestone-associated *Boesenbergia* population from Loei Province represents a distinct species rather than an environmentally induced form or an extreme variant of *B. rotunda* sensu lato. Therefore, the present study evaluates the limestone-associated *Boesenbergia* population from Loei Province as a morphology-based taxonomic hypothesis, using repeated observations of living material, herbarium and literature comparisons, and focused phenetic analyses of morphologically similar taxa. In addition, a diagnostic character matrix and a focused phenetic similarity analysis of selected morphologically similar taxa lacking an androecial cup were used to evaluate the morphological distinctiveness of the Loei population. The aims of this study are to describe and illustrate the new species, assess its diagnostic characters using comparative morphological and phenetic evidence, and discuss its limestone microhabitat and preliminary conservation status.

## 2. Materials and Methods

### 2.1. Field Observations, Living Material, and Ex Situ Cultivation

**Figure 1:**
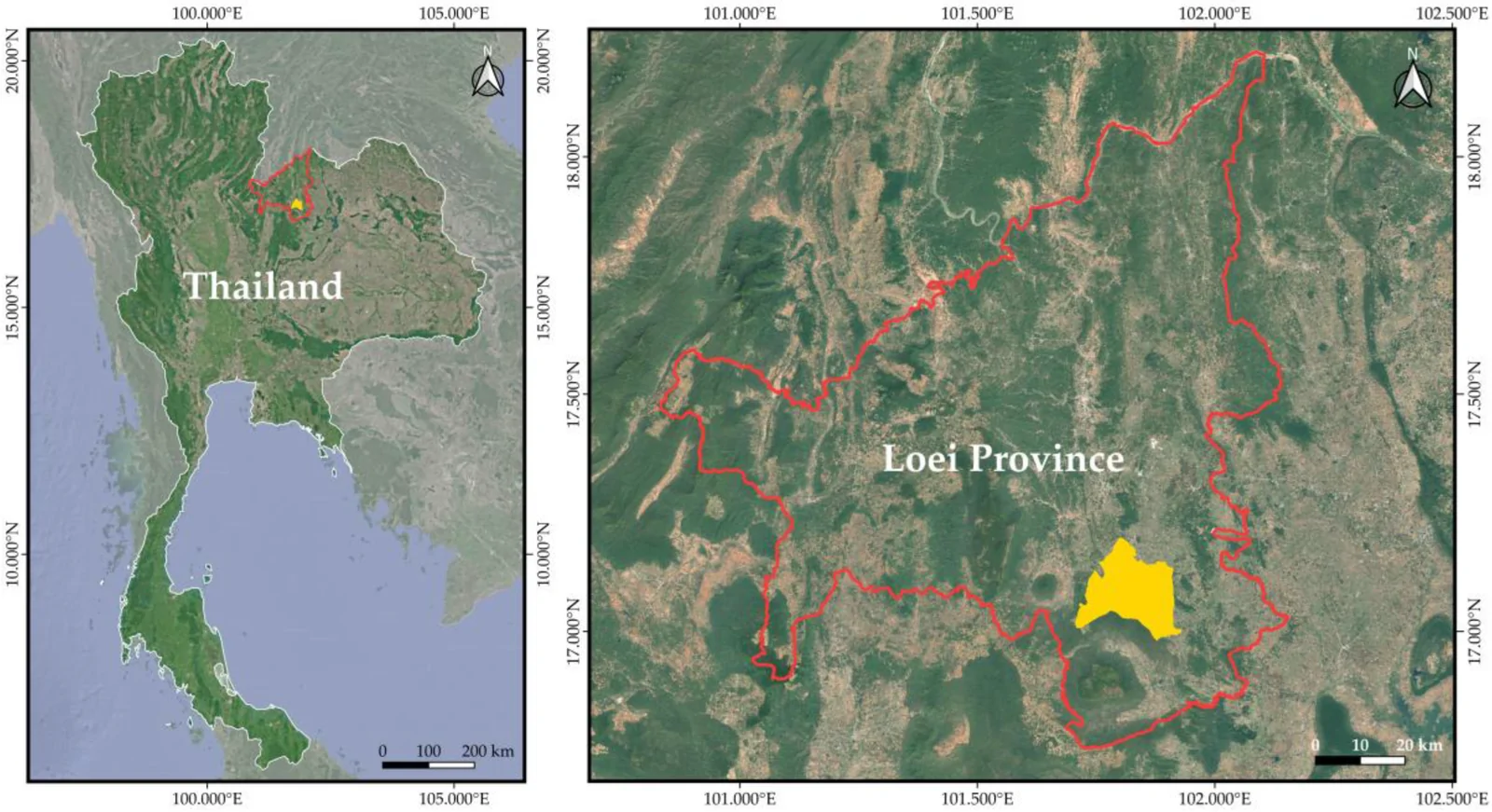
Location of the study area of *Boesenbergia lamudiae* Boonma, Saensouk & P.Saensouk sp. nov. in Nong Hin District, Loei Province, northeastern Thailand. The left map indicates the position of Loei Province within Thailand, with the yellow area representing the study district. The right map shows the boundary of Loei Province in red and the study area in Nong Hin District in yellow (map created with QGIS program ver. 3.34, geographic system ID: WGS 84, EPSG 4326).

The morphological description of the new species was based primarily on living flowering plants observed in a natural population in a limestone-associated habitat in Loei Province, northeastern Thailand (Figure 1).

The population was first documented in 2019, and additional field observations were made to confirm the persistence of diagnostic characters in the natural habitat. Measurements were obtained from 20 mature flowering individuals observed in situ to ensure consistency in character assessment. Because the population at the study site was estimated to comprise fewer than 50 individuals, collection was kept to a minimum. Only five rhizomes were collected for ex situ cultivation to avoid excessive disturbance to the natural population.

The collected rhizomes were cultivated at Brio Botanical Research Garden, Nakhon Nayok, Thailand. Flowering plants derived from the type locality were monitored under cultivation to evaluate whether diagnostic characters remained stable outside the natural habitat. Attention was given to the persistence of floral characters, including calyx length and indumentum, calyx apex shape, labellum shape and colour pattern, anther crest morphology, and ovary indumentum. These observations were used to assess character stability but were not treated as separate morphometric samples from the natural population. The study site is located outside a protected area, and no specific permits were required for plant collection under local regulations. Voucher specimens of the studied material were deposited at the Vascular Plant Herbarium, Mahasarakham University (VMSU).

### 2.2. Herbarium Study and Comparative Taxonomic Sources

Herbarium specimens were studied through a combination of direct examination and digital resources. Accessible collections were investigated in person, whereas additional material was examined using high-resolution images from online repositories, including the Chinese Virtual Herbarium [18]. Specimens examined on-site were from the following herbaria: B, BK, BKF, BM, C, CAL, DMSC, E, HN, HNPM, HNU, IBK, IMDY, KKU, L, P, QBG, SGN, SING, VMSU, VNM, and VNMN. Specimens examined exclusively through digital images were from A, AAU, BR, HITBC, K, NY, RAF, TNS, and US. Herbarium acronyms follow the Index Herbariorum [19].

Type material, protologues, and published descriptions were consulted where available. Comparative assessments were conducted using published descriptions [2,5,6,7,8,9,10,11,12,20,21,22,23,24,25,26,27,28,29,30,31,32,33,34,35,36,37,38,39], herbarium specimens, and field observations of morphologically similar taxa within *Boesenbergia*. Particular emphasis was placed on taxa lacking an androecial cup and on species or previously described forms morphologically similar to the Loei population, including *B. burmanica*, *B. rotunda* sensu lato, *B. meghalayensis*, *B. baimaii* and *B. isanensis*.

These taxa and published regional descriptions were selected because they share key features relevant to the delimitation of the Loei population, especially the terminal inflorescence enclosed within the leaf sheaths, absence of an androecial cup, and overall similarity to the *B. rotunda* sensu lato complex. The comparison was therefore designed as a focused taxonomic assessment rather than a genus-wide revision of *Boesenbergia*.

### 2.3. Morphological Measurements and Terminology

Measurements of vegetative and reproductive structures were taken from fresh material using a standard ruler and a digital vernier caliper (Mitutoyo Corporation, Kawasaki, Japan). Fine morphological features, including surface indumentum, calyx structure, labellum morphology, anther crest, stigma, ovary indumentum, and inflorescence structure, were examined using a stereomicroscope (Stemi 2000-C; Carl Zeiss, Oberkochen, Germany). Morphological terminology follows Beentje [40]. Nomenclature was verified using Plants of the World Online [1] and the International Plant Names Index [41].

### 2.4. Comparative Morphological Analysis

A comparative morphological analysis was conducted using both quantitative and qualitative characters. Quantitative characters, such as flower length, calyx length, floral tube length, labellum size, lateral staminode size, anther crest size, ovary size, and epigynous gland length, were summarized as measurement ranges. Qualitative characters, such as calyx indumentum, calyx apex shape, labellum saccation, labellum colour pattern, anther crest shape, stigma morphology, ovary indumentum, and habitat, were evaluated as diagnostic character states. Because measurements of related taxa were mostly available as ranges from protologues and published descriptions rather than as individual-level raw data, multivariate analyses requiring raw individual measurements, such as PCA, were not performed. Instead, comparative evidence was summarized using a diagnostic character matrix and selected range comparisons. This approach allowed for direct comparison between the newly collected living material and published taxonomic evidence without treating literature ranges as individual observations.

### 2.5. Phenetic Analysis

To evaluate overall morphological similarity among *Boesenbergia lamudiae* and relevant related taxa, a focused phenetic similarity analysis was performed using 25 selected vegetative and floral characters. The analysis was not designed as a genus-wide assessment of *Boesenbergia*. Instead, it was restricted to selected morphologically similar taxa lacking an androecial cup, with particular emphasis on taxa associated with the *B. rotunda* sensu lato complex and species relevant to the delimitation of *B. lamudiae*. Published regional descriptions of *B. rotunda* sensu lato were treated as separate operational units to avoid obscuring regional morphological variation within a single polymorphic taxon. This treatment was used only for comparative phenetic purposes and does not imply taxonomic recognition of these operational units as separate taxa.

Habitat and distributional characters were excluded from the phenetic matrix to avoid ecological or geographical bias in the clustering. Quantitative characters were converted into discrete character states, whereas qualitative characters were coded as categorical states. A similarity dendrogram was generated using unweighted pair group method with arithmetic mean (UPGMA) [42] based on Gower distance [43]. Principal coordinates analysis (PCoA) was also performed using the same Gower distance matrix to visualize overall morphological separation among the operational units. The analysis was conducted using PAST software version 4.15 [44]. The resulting dendrogram and PCoA were interpreted only as phenetic summaries of morphological similarity and were not used as evidence of phylogenetic relationships or evolutionary independence. The character definitions and coded character-state matrix are provided in Table A1 and Table A2, respectively.

### 2.6. Microhabitat and Population Observations

Microhabitat observations were recorded at the type locality, including substrate, elevation, shading, limestone association, and the approximate number of observed individuals. The habitat was characterized by shaded limestone-associated conditions with sandy loam soil mixed with limestone rocks at approximately 420–450 m above sea level (a.s.l.). The observed population size was estimated to be fewer than 50 individuals. These field observations were used to provide ecological context for the new species and to support discussion of limestone microhabitats as potential reservoirs of localized Zingiberaceae diversity.

### 2.7. Preliminary Conservation Assessment

The conservation status of the new species was preliminarily assessed following the IUCN Red List Categories and Criteria [45]. Because the species is currently known only from the type locality, and because adjacent limestone habitats have not yet been sufficiently surveyed, the extent of occurrence, area of occupancy, number of locations, population trend, and continuing threats remain insufficiently known. The assessment is therefore treated as provisional.

### 2.8. Figure Preparation

All photographic plates and figure compositions were prepared using Pixelmator Pro (version 3.6.5, “Archipelago”; Pixelmator Team, Vilnius, Lithuania) on a MacBook Pro (13-inch, M1, 2020; Apple Inc., Cupertino, CA, USA). Scientific illustrations were digitally created using an iPad Air (5th generation; Apple Inc.) running iPadOS 17.5.1. All figures were prepared and drawn by the first author.

## 3. Results

Field observations, ex situ cultivation, and comparative morphological assessment support the recognition of the limestone-associated *Boesenbergia* population from Loei Province as a distinct species. The plants consistently exhibited a stable combination of vegetative and floral characters in both the natural population and cultivated material derived from the type locality. Among the taxa examined, the Loei population is morphologically most similar to *B. burmanica* and resembles *B. rotunda* sensu lato in having a terminal inflorescence enclosed within the leaf sheaths and lacking an androecial cup. However, it differs from these and other morphologically similar taxa by its unusually long pubescent calyx with an acute to acuminate apex, non-saccate obovate labellum with a sharply delimited deep reddish to maroon transverse band across the throat, larger recurved anther crest, and pubescent ovary. The diagnostic characters observed in living flowering material remained stable under ex situ cultivation and did not correspond to any previously described species. Therefore, the Loei population is described here as a new species, *B. lamudiae* Boonma, Saensouk & P.Saensouk.

### 3.1. Taxonomic Treatment of New Species

***Boesenbergia lamudiae*** **Boonma, Saensouk & P.Saensouk, sp. nov.**

**Type:** THAILAND, Northeastern, Loei Province, Nong Hin District, c. 425 m elevation, 7 July 2019, *T.Boonma 77* (holotype VMSU).

**Figure 2:**
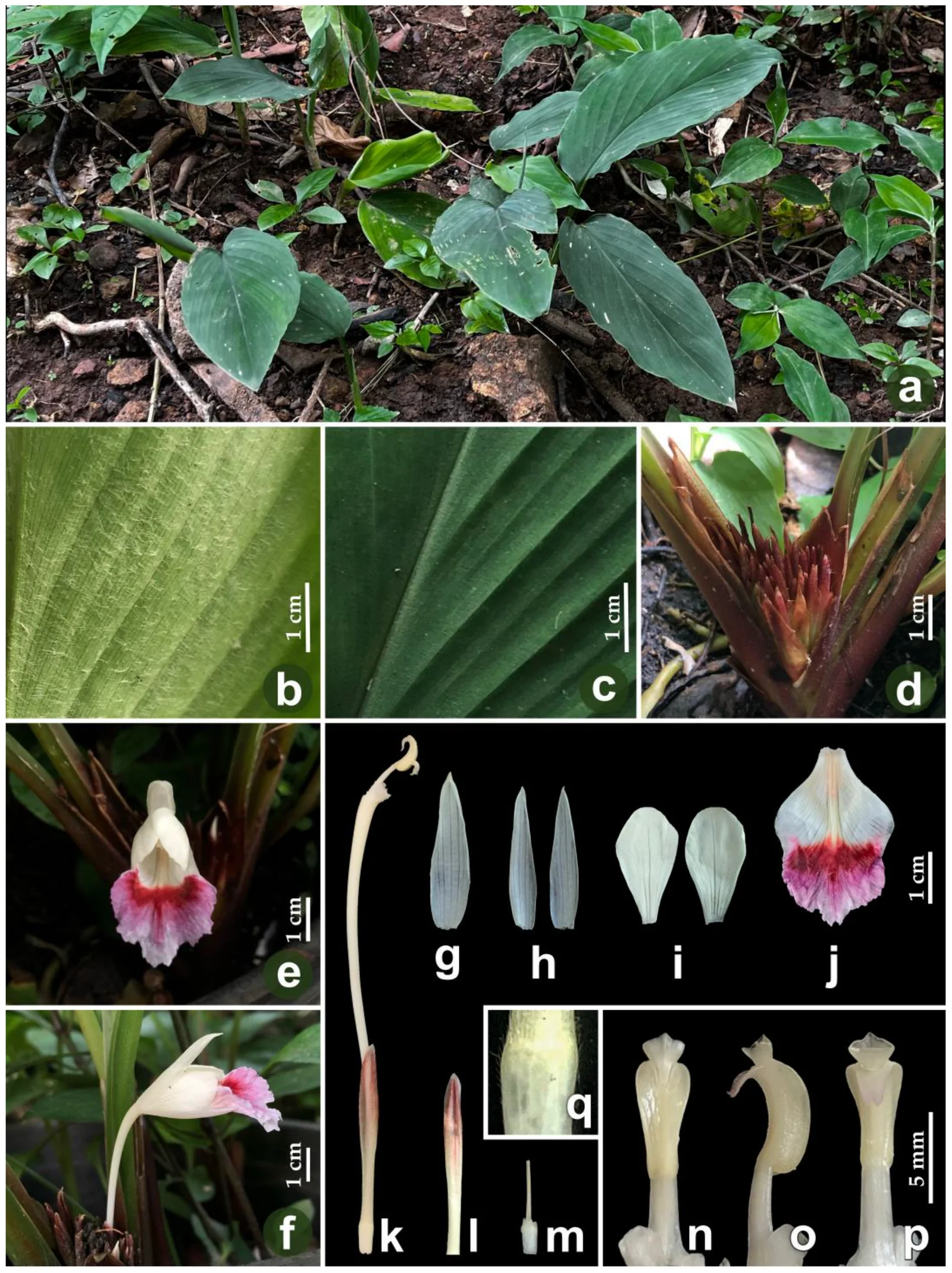
*Boesenbergia lamudiae* Boonma, Saensouk & P.Saensouk, sp. nov.: (**a**) Habit in natural habitat. (**b**) Abaxial leaf surface. (**c**) Adaxial leaf surface. (**d**) Terminal inflorescence enclosed within the leaf sheaths. (**e**) Front view of flower. (**f**) Side view of flower. (**g**) Dorsal corolla lobe. (**h**) Lateral corolla lobes. (**i**) Lateral staminodes. (**j**) Labellum. (**k**) Floral tube with stamen, calyx, and ovary. (**l**) Calyx. (**m**) Ovary with epigynous glands. (**n**–**p**) Front, lateral, and back views of stamen. (**q**) Close up of pubescent ovary. Photographs by Thawatphong Boonma.

**Figure 3:**
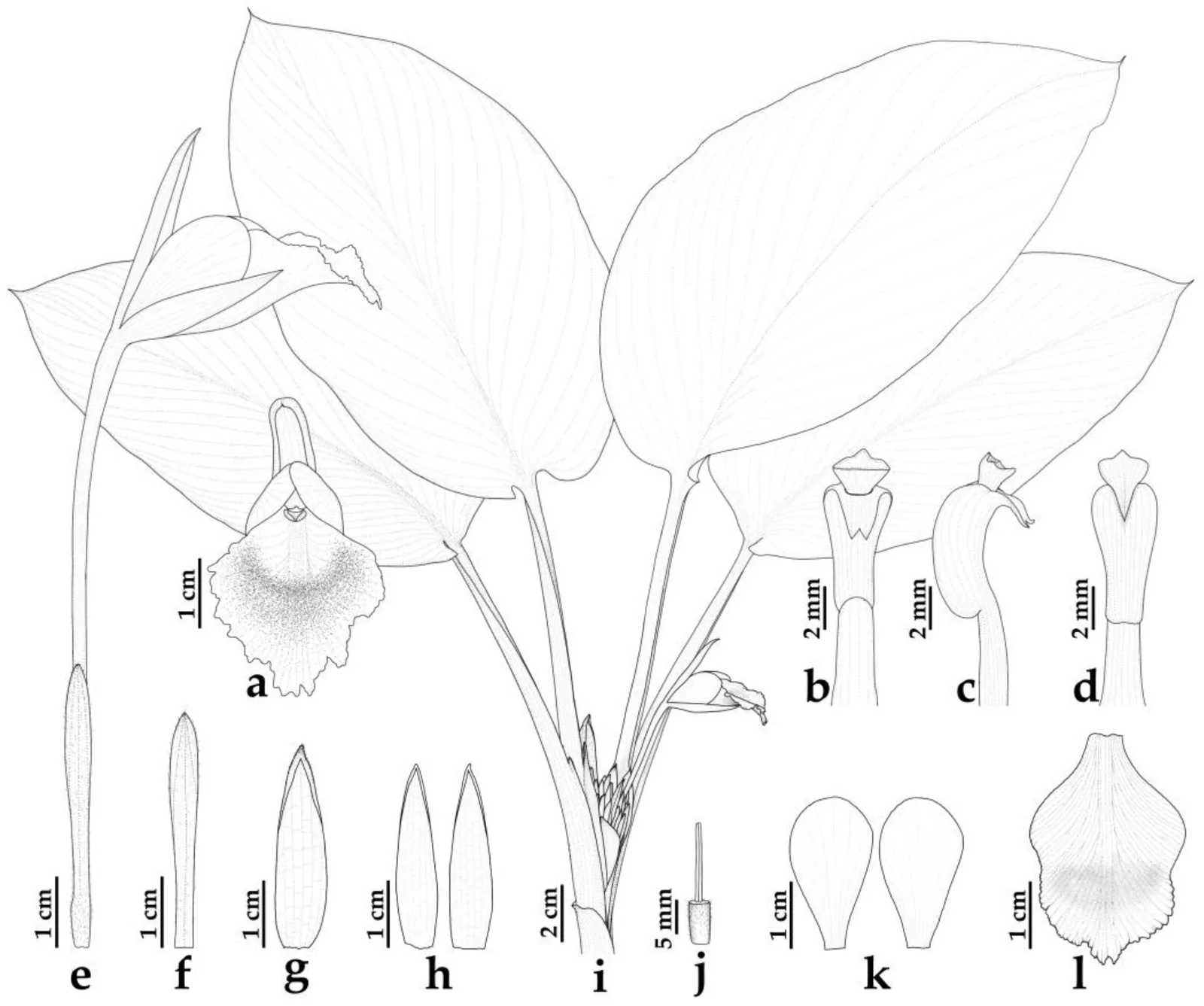
*Boesenbergia lamudiae* Boonma, Saensouk & P.Saensouk, sp. nov.: (**a**) Front view of flower. (**b**–**d**) Back, lateral, and front views of stamen. (**e**) Side view of flower. (**f**) Calyx. (**g**) Dorsal corolla lobe. (**h**) Lateral corolla lobes. (**i**) Habit. (**j**) Ovary with epigynous glands. (**k**) Lateral staminodes. (**l**) Labellum. Drawn by Thawatphong Boonma.

**Diagnosis.** *Boesenbergia lamudiae* is morphologically most similar to *B. burmanica* Boonma, P.Saensouk & Saensouk in having a terminal inflorescence enclosed within the leaf sheaths, pubescent to puberulous vegetative parts, puberulent to pubescent calyx, absence of an androecial cup, non-saccate labellum, recurved anther crest, and cup-shaped stigma, but differs by its larger laminae, mostly 30–42 × 8–15 cm, broadly elliptic to ovate-elliptic or sometimes slightly oblong-elliptic (vs. 10–34 × 4–7.5 cm, narrowly elliptic), more flowers per spike (15–20 vs. 9–10), maroon narrowly lanceolate to lanceolate bracts (vs. pale green oblong bracts), longer flowers (10.5–12 cm vs. 8–9 cm), longer calyx with an acute to acuminate apex (3.2–3.6 cm long vs. 2.2–2.5 cm long and bifid), longer floral tube (7.8–8.5 cm vs. 5.5–5.8 cm), larger white glabrous lateral staminodes (2.2–2.5 × 1.2–1.4 cm vs. 1.4–1.5 × 0.8–0.9 cm, sparsely puberulous), obovate labellum with a distinct zonal colour pattern, the basal portion white to creamy white with a very pale pinkish tint along the central basal region and midline, the throat with a sharply delimited deep reddish to maroon transverse band, and the distal portion pinkish purple to pale pink, darker toward the centre and apex, with conspicuously darker veins, rarely with small reddish spots (vs. oblanceolate labellum, white with bright red towards the apex and reddish spots in the midlobe), larger anther crest (ca. 3 × 2.5 mm vs. ca. 1 × 1 mm), longer epigynous glands (10–12 mm vs. 7.4–8 mm), and larger pubescent ovary (5–7 × 2.5–3 mm vs. ca. 3 × 2 mm, glabrous). It is also distinguished from *B. rotunda* sensu lato by its abaxially arachnoid laminae, longer pubescent calyx with an acute to acuminate apex, non-saccate obovate labellum with a sharply delimited transverse colour band, longer epigynous glands, pubescent ovary, and apparently restricted limestone-associated habitat in Loei Province, northeastern Thailand (Table 1, Figure 2 and Figure 3).

**Description:** Perennial rhizomatous herb, 35–50 cm tall. Primary rhizome ovoid, 1.5–2 cm in diameter, externally brown, internally yellow, aromatic; branches opposite, producing new shoots and forming a loose clump. Roots fibrous with ovoid to ellipsoid tubers, tapering distally. Pseudostem 10–18 cm tall; sheath distichous, maroon to russet, sometimes green distally, pubescent. Leaves (3–)4–6 per pseudostem; ligule bilobed, 1.5–1.7 cm long, lobes triangular, apex acute, russet; petiole 8–20 cm long, canaliculate, green, puberulous; lamina broadly elliptic to ovate-elliptic, sometimes slightly oblong-elliptic, subcoriaceous, (15–)30–42 × (6–)8–15 cm, green, adaxially glabrous, minutely punctate, slightly glossy, veins prominent; abaxially arachnoid, base attenuate to narrowly cuneate, slightly decurrent onto the petiole, apex acute to shortly acuminate, often minutely mucronulate, margin undulate. Inflorescence terminal, 5–9 cm long, peduncle 1–3 cm long, cylindrical, whitish tinged maroon distally, puberulous, enclosed within the innermost leaf sheaths; spike 4–6 cm long, with 15–20 flowers, opening successively, usually one at a time, rarely two. Bracts narrowly lanceolate to lanceolate, 2.5–4.2 × 0.5–1 cm, apex acute to acuminate, densely imbricate, maroon, puberulent. Bracteoles lanceolate, 2.0–3.8 × 0.4–0.8 cm, similar in colour to bracts but more translucent, apex acute to acuminate, puberulent. Flowers 10.5–12.0 cm long. Calyx tubular 3.2–3.6 cm long, 3.0–3.5 mm in diameter, membranous, apex acute to acuminate, white with a reddish tinge distally, pubescent, with a unilateral slit up to 8 mm long. Floral tube cylindrical, 7.8–8.5 cm long, white, puberulous; dorsal corolla lobe lanceolate, 2.8–3.0 × 0.6–0.7 cm, apex acuminate with mucronate tip, hooded, white, sparsely puberulent at the tip; lateral corolla lobes lanceolate, 2.5–2.6 × 0.50–0.52 cm, apex acute, slightly hooded, white, sparsely puberulous. Lateral staminodes 2, obovate, 2.2–2.5 × 1.2–1.4 cm, white, apex rounded, margin entire, glabrous. Labellum obovate, not saccate, 3.2–3.5 × 2.0–2.2 cm, forming a distinct zonal colour pattern; basal portion white to creamy white, with a very pale pinkish tint along the central basal region and midline; throat with a sharply delimited deep reddish to maroon transverse band, extending laterally across the blade; distal portion pink to pale pink, darker toward the centre and apex, with veins conspicuously darker, rarely with small reddish spots; apex distinctly emarginate; margins strongly undulate to crisped. Stamen 1, 11–12 mm long; filament 6.2–6.5 × 2 mm, white, glabrous; anther 6.5–7.2 × 2–3 mm, cream; anther crest triangular, c. 3 mm long, c. 2.5 mm wide at base, recurved backwards, cream at base with a pinkish tinge distally, apex emarginate. Style filiform, 8.5–8.7 cm long, white; stigma cup-shaped, ciliate, white. Epigynous glands two, 10–12 mm long, linear, pale cream-colored. Ovary cylindrical, 5–7 × 2.5–3 mm, axile placentation, trilocular, pubescent. Fruit not seen.

**Distribution and habitat:** *Boesenbergia lamudiae* is currently known only from its type locality in Loei Province, Thailand. It grows on sandy loam soil mixed with limestone rocks in a shady area, at an elevation of approximately 420–450 m above sea level.

**Phenology:** Flowering in June to September, fruit not seen.

**Etymology:** The specific epithet “*lamudiae*” honors the late Lamud Boonma (3 April 1969–29 March 2026), in recognition of her valuable contributions and support to the first author in studying the diversity of Zingiberaceae in Thailand.

**Additional specimens examined (paratype):** THAILAND, Northeastern, Loei Province, Nong Hin District, c. 430 m elevation, 7 July 2020, *T.Boonma 2078* (VMSU).

**Utilization:** Occasionally cultivated as an ornamental; no other uses recorded.

**Key to *Boesenbergia lamudiae* and selected morphologically similar taxa lacking an androecial cup**
1a.Epigynous glands > 10 mm long; Calyx 3.2–3.6 cm long
*B. lamudiae*
1b.Epigynous glands < 10 mm long; Calyx ≤ 2.5 cm long22a.Leaves narrowly elliptic, adaxial surface puberulent
*B. burmanica*
2b.Leaves elliptic-oblong, adaxial surface glabrous33a.Calyx sparsely hairy or pubescent43b.Calyx glabrous54a.Leaf midrib green; Labellum pale pink in the apical part with deep pink to magenta patches around the throat, and cream in the basal half
*B. baimaii*
4b.Leaf midrib dark red; Labellum red in the apical half and cream in the basal half
*B. isanensis*
5a.Staminodes white; Labellum white with maroon towards tip and maroon spots in the throat
*B. meghalayensis*
5b.Staminodes pale pink to pink; Labellum deep pink towards tip and white with crimson spots and bands radiating laterally in the throat
*B. rotunda*



### 3.2. Phenetic Similarity Analysis

**Figure 4:**
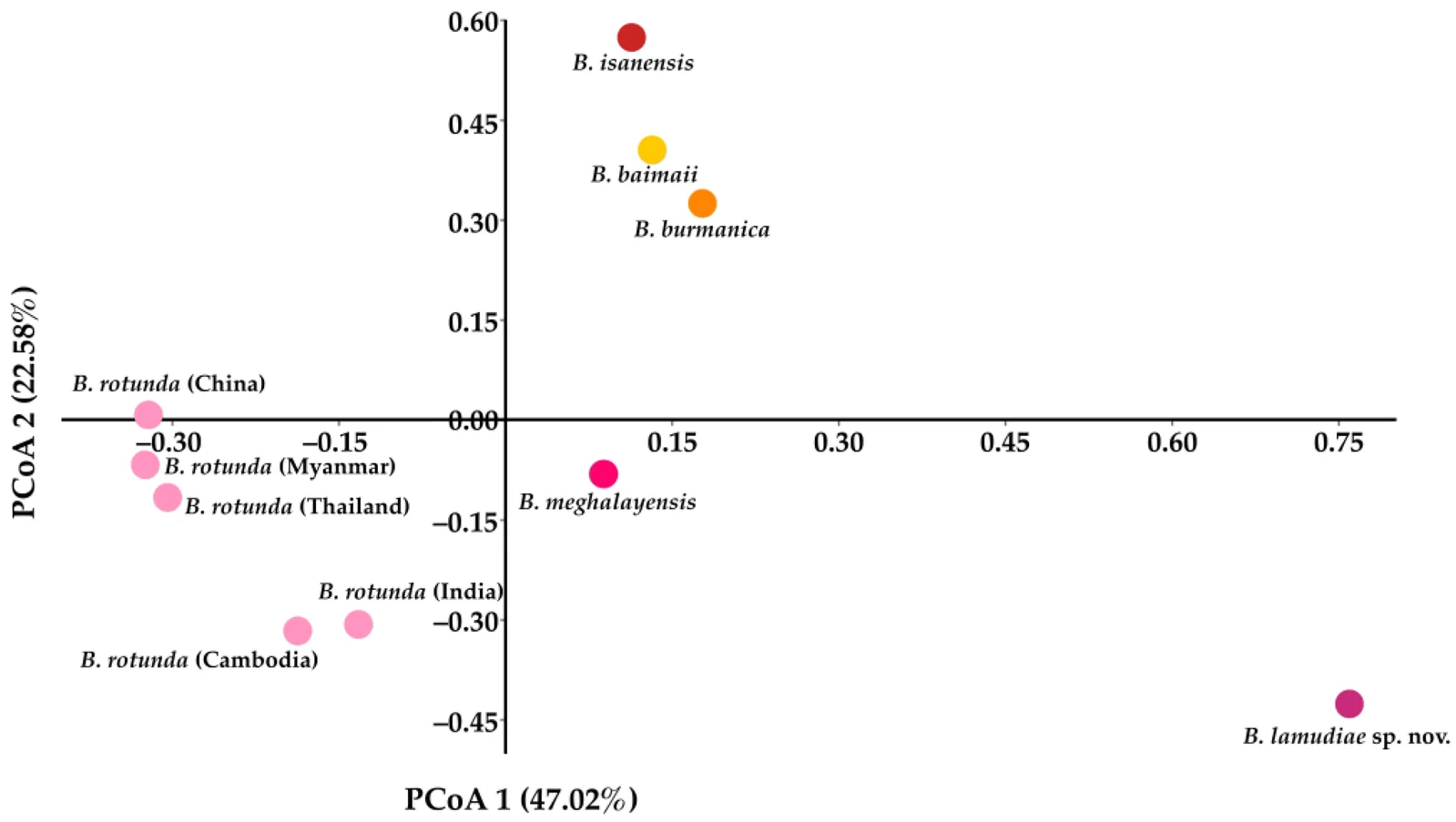
Principal coordinates analysis (PCoA) scatter plot based on Gower distance calculated from 25 selected morphological characters of *Boesenbergia lamudiae* and selected morphologically similar taxa lacking an androecial cup. The first two coordinate axes explain 69.60% of the total morphological variation, with PCoA 1 accounting for 47.02% and PCoA 2 for 22.58%.

The PCoA based on Gower distance separated *Boesenbergia lamudiae* from all compared operational units (Figure 4). The first two coordinate axes explained 69.60% of the total morphological variation, with PCoA 1 accounting for 47.02% and PCoA 2 for 22.58%. *Boesenbergia lamudiae* was positioned as an isolated point on the positive side of PCoA 1, clearly apart from *B. burmanica*, *B. meghalayensis*, and all regional operational units of *B. rotunda* sensu lato.

**Figure 5:**
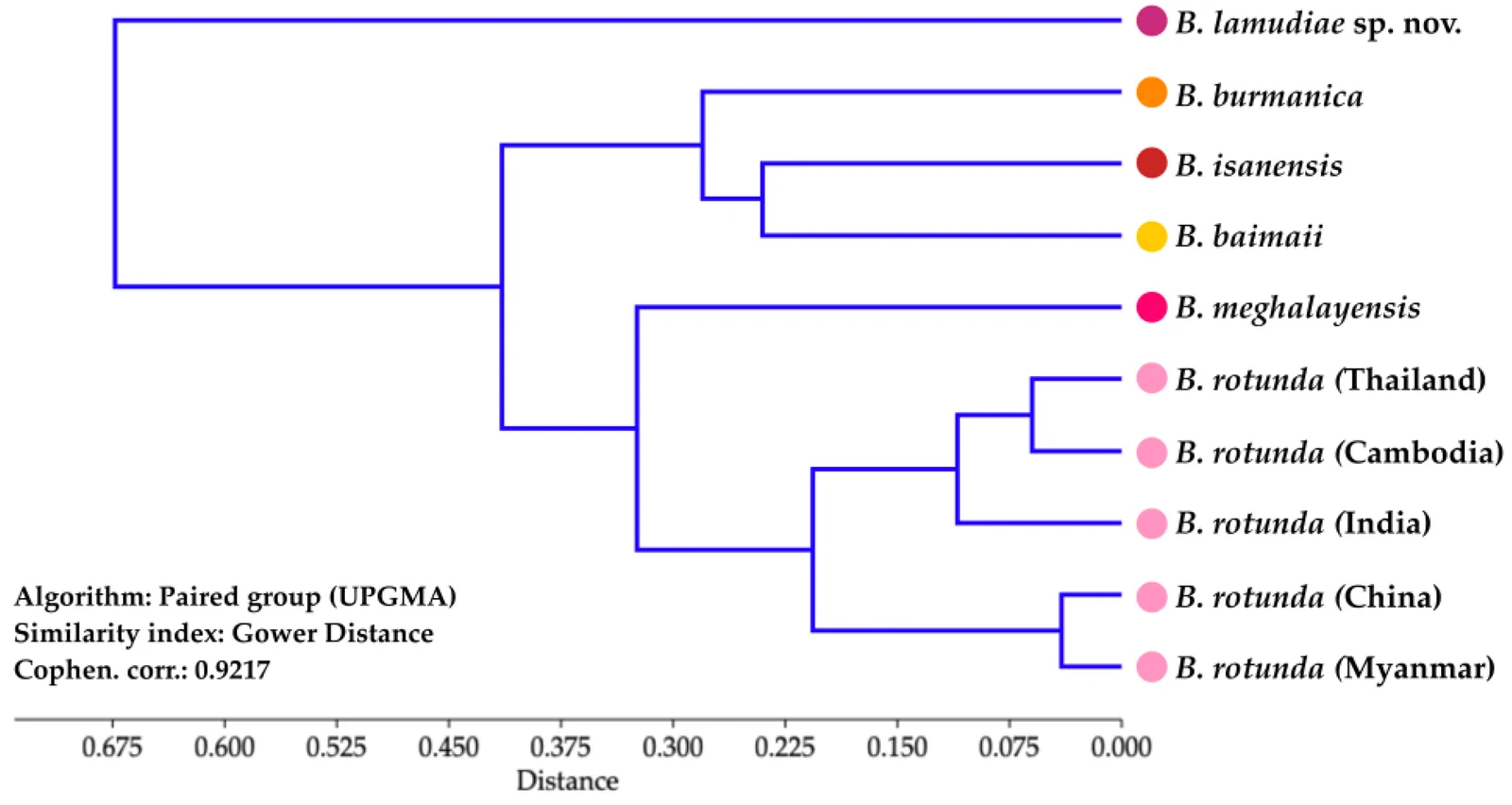
UPGMA dendrogram based on Gower distance calculated from 25 selected morphological characters of *Boesenbergia lamudiae* and selected morphologically similar taxa lacking an androecial cup. The cophenetic correlation coefficient is 0.9217. The dendrogram is interpreted as a phenetic summary of morphological similarity and not as a phylogenetic hypothesis.

The UPGMA dendrogram based on the same Gower distance matrix also separated *B. lamudiae* from the remaining operational units (Figure 5). The dendrogram showed a high cophenetic correlation coefficient of 0.9217, indicating that the clustering pattern represented the original distance matrix well. The remaining operational units were arranged into subgroups corresponding broadly to *B. burmanica*, the protologue-based operational units of *B. baimaii* and *B. isanensis*, *B. meghalayensis*, and the regional operational units of *B. rotunda* sensu lato. These results support the morphological distinctiveness of *B. lamudiae* from the selected comparable taxa.

### 3.3. Microhabitat and Preliminary Conservation Status

*Boesenbergia lamudiae* was observed in a shaded limestone-associated habitat in Nong Hin District, Loei Province, northeastern Thailand. Plants grew in sandy loam soil mixed with limestone rocks at approximately 420–450 m above sea level, in a microhabitat characterized by partial shade and limestone-derived substrate. The species was found growing in a small and localized population, and fewer than 50 mature individuals were observed at the only currently known locality.

The known population was first observed in 2019 and revisited in 2020 and 2025. During these revisits, no marked decline in the natural population was observed; however, the population also did not show a notable increase and remains very small. At present, there is no evidence of collection for trade or local harvesting pressure, probably because the species is not yet widely known or commonly used when compared with other economically important species of *Boesenbergia*. Nevertheless, the known population remains highly restricted, and adjacent limestone habitats have not yet been sufficiently surveyed.

Based on the currently known population, *B. lamudiae* is provisionally assessed as Critically Endangered (CR) under criterion D, following the IUCN Red List Categories and Criteria [45], as fewer than 50 mature individuals were observed at the only known locality. The species does not currently have sufficient data for assessment under criterion A, because population reduction has not been documented during repeated observations. Criteria B and C are also not applied here because the extent of occurrence (EOO), area of occupancy (AOO), number of locations, and evidence of continuing decline remain inadequately known or have not been demonstrated. Criterion E is not applicable, as no quantitative extinction-risk analysis has been conducted. Therefore, this assessment should be regarded as preliminary pending further field surveys in the surrounding limestone areas.

## 4. Discussion

### 4.1. Taxonomic Significance of Boesenbergia lamudiae

The recognition of *Boesenbergia lamudiae* as a new species is supported by a combination of vegetative and floral characters that is not found in the selected morphologically similar taxa. The species shares with *B. rotunda* sensu lato and several related taxa a terminal inflorescence enclosed within the leaf sheaths and the absence of an androecial cup, a character combination that can make species delimitation difficult when incomplete material is available [2,4,9,17,20,21,28,39]. However, *B. lamudiae* differs consistently in having broadly elliptic to ovate-elliptic laminae with an arachnoid abaxial surface, maroon lanceolate bracts, a long pubescent calyx with an acute to acuminate apex, a non-saccate obovate labellum with a sharply delimited maroon transverse colour zone, a larger anther crest, longer epigynous glands, and a pubescent ovary. Among these characters, the arachnoid abaxial lamina surface, calyx exceeding 3.0 cm in length, and pubescent ovary are particularly useful for separating *B. lamudiae* from the compared taxa.

The closest morphological comparison is with *B. burmanica*, which also has relatively large flowers and lacks an androecial cup [11]. Nevertheless, *B. lamudiae* differs from *B. burmanica* by its broader laminae, arachnoid abaxial lamina surface, maroon rather than pale green to whitish bracts, higher number of flowers per spike, longer calyx, acute to acuminate rather than bifid calyx apex, longer floral tube, larger anther crest, longer epigynous glands, and pubescent ovary. These differences involve several independent vegetative and floral structures and are therefore unlikely to represent only minor intraspecific variation.

The comparison with *B. rotunda* sensu lato is also important because this species has been interpreted broadly in regional floristic treatments and taxonomic accounts [2,4,9,17,28,39]. Across the compared descriptions of *B. rotunda* sensu lato, the lamina is usually glabrous, glabrescent, or only finely hairy beneath, the calyx is shorter and glabrous, the labellum is slightly concave or slightly saccate, and the ovary is glabrous or nearly glabrous [2,4,9,17,28,39]. In contrast, *B. lamudiae* has an arachnoid abaxial lamina surface, a markedly longer pubescent calyx, a non-saccate obovate labellum with a sharply defined transverse colour zone, and a pubescent ovary. These features place *B. lamudiae* outside the observed morphological range of the selected regional operational units of *B. rotunda* sensu lato.

*Boesenbergia rotunda* sensu lato is treated here conservatively and only as a comparative framework. The present study does not attempt to revise the *B. rotunda* complex, because the taxonomic limits of some previously described taxa and regional forms require further reassessment based on well-documented living material, dissected floral characters, and broader population-level sampling. Therefore, the use of *B. rotunda* sensu lato in this study should not be interpreted as a taxonomic decision on those names, but as a broad comparative context for evaluating the distinctiveness of *B. lamudiae*. Although several vegetative indumentum characters partly overlap within this broad circumscription, *B. lamudiae* is not distinguished by any single variable character alone. Rather, it is diagnosed by a stable combination of independent vegetative and floral characters, especially the arachnoid abaxial lamina surface, markedly longer pubescent calyx, acute to acuminate calyx apex, non-saccate obovate labellum with a sharply delimited transverse colour zone, longer epigynous glands, and pubescent ovary.

The protologue-based operational units corresponding to *B. isanensis* and *B. baimaii* are also useful for comparison because they represent morphologically differentiated elements that have previously been associated with the broader *B. rotunda* complex. The material described as *B. isanensis* differs from *B. lamudiae* by its shorter calyx, different leaf indumentum, green bracts, shorter epigynous glands, and glabrous ovary [20]. The material described as *B. baimaii* differs by its smaller habit, narrower laminae, glabrous to glabrescent vegetative parts, shorter calyx, different labellum colour pattern, smaller anther crest, shorter epigynous glands, and glabrous ovary [21]. Therefore, the diagnostic characters of *B. lamudiae* are not limited to labellum colour but involve a broader set of stable morphological differences.

### 4.2. Phenetic Evidence and Interpretation

The phenetic analyses provide additional support for the morphological separation of *B. lamudiae*. Both the PCoA and UPGMA analyses, based on Gower distance calculated from 25 selected morphological characters, separated *B. lamudiae* from all compared operational units. This separation is consistent with the diagnostic discontinuities observed in the morphology, particularly in lamina indumentum, calyx length and indumentum, labellum structure and colour pattern, epigynous gland length, and ovary indumentum.

The use of Gower distance is appropriate for the present dataset because the character matrix includes categorical and multistate morphological data rather than only continuous measurements [43,46]. The resulting ordination and clustering should not be interpreted as a phylogenetic hypothesis, but as a phenetic summary of morphological similarity among the selected taxa. In this context, the isolated position of *B. lamudiae* is taxonomically meaningful because it agrees with the qualitative morphological comparisons and the diagnostic key.

The scope of the phenetic analysis was deliberately restricted to selected morphologically similar taxa lacking an androecial cup, especially taxa relevant to the delimitation of *B. lamudiae* and taxa associated with the *B. rotunda* sensu lato complex. This restricted sampling avoids the overinterpretation that would result from treating the analysis as a genus-wide assessment. It also allows regional descriptions of *B. rotunda* sensu lato to be compared separately, which is useful because the name has been applied to morphologically variable material across different parts of its range [2,4,9,17,20,21,28,39]. The results indicate that *B. lamudiae* does not fall within the morphological space occupied by these selected comparable taxa.

### 4.3. Value of Living Material in Species Delimitation

The present study reinforces the importance of living material in the taxonomy of *Boesenbergia*. Several diagnostic characters in the genus are difficult to interpret from dried specimens alone, including labellum colour pattern, floral texture, bract colour, indumentum, anther crest morphology, and the three-dimensional form of the labellum [17,23,25,31,39]. In *B. lamudiae*, the sharply delimited maroon transverse colour zone of the labellum, the arachnoid abaxial lamina surface, and the pubescent ovary were confirmed from living plants and supported by repeated observations. These characters may become obscure, distorted, or partly lost in herbarium specimens.

The interpretation of lateral staminode colour in regional accounts of *B. rotunda* provides a clear example of this difficulty. In some treatments, the lateral staminodes are described as white; however, the accompanying illustrations show them as pale pink to pink, sometimes with a white basal portion and a pink distal portion [4,28]. For this reason, the staminode colour state in the present analysis was coded according to the condition clearly visible in the published illustrations when it conflicted with the written description. This approach reduces the risk of underestimating colour variation caused by inconsistencies between descriptions and illustrations.

This issue is particularly relevant to the *B. rotunda* sensu lato complex, in which taxonomic interpretation has often relied on regional descriptions, herbarium material, illustrations, and cultivated or wild-collected plants [2,4,9,17,28,39]. Differences in preservation, flowering stage, colour documentation, and descriptive emphasis can affect the interpretation of floral characters. For this reason, the present study integrates living observations, herbarium comparisons, published descriptions, illustrations, and a coded morphological matrix. Such an approach provides a more reliable basis for evaluating whether the Loei population represents a distinct taxon.

### 4.4. Limestone Microhabitat and Conservation Implications

*Boesenbergia lamudiae* is currently known only from a shaded limestone-associated habitat in Nong Hin District, Loei Province, northeastern Thailand. The plants grow in sandy loam soil mixed with limestone rocks at approximately 420–450 m above sea level. Limestone landscapes in mainland Southeast Asia are known to support localized plant diversity because discontinuous outcrops create small habitat islands with distinct substrate, moisture, and shade conditions [47,48,49]. Such habitats may provide suitable microenvironments for narrowly distributed Zingiberaceae, but they are also vulnerable to disturbance because populations are often small and spatially restricted [10,17].

The only known population of *B. lamudiae* contains fewer than 50 mature individuals. Repeated observations did not show a marked decline, and there is currently no evidence of collection for trade or local harvesting. Nevertheless, the species remains conservation-relevant because its known population is extremely small and its distribution is highly restricted. The absence of observed decline should not be interpreted as evidence of security, because adjacent limestone habitats have not yet been sufficiently surveyed. Although the minimum AOO can be estimated as 4 km^2^ based on a single occupied 2 × 2 km grid cell, the full distribution, EOO, number of occupied grid cells, population trend, and potential threats remain insufficiently known. 

Based on the available data, *B. lamudiae* is provisionally assessed as Critically Endangered (CR) under criterion D, following the IUCN Red List Categories and Criteria [45], because fewer than 50 mature individuals were observed at the only currently known locality. Recent population-based assessments of narrowly distributed threatened plants have highlighted the value of combining mature-individual counts, locality information, AOO/EOO estimation, habitat observations, and threat documentation [50]. In the present case, the minimum AOO is estimated as 4 km^2^ based on a single occupied 2 × 2 km grid cell. However, Criterion B2 is not applied here because a continuing decline in AOO, EOO, habitat quality, number of locations or subpopulations, or number of mature individuals has not yet been demonstrated. Therefore, the present assessment should be regarded as provisional, and further surveys and population monitoring in surrounding limestone areas are needed to determine whether additional populations exist, whether any continuing decline can be inferred or projected, and whether Criterion B2 may become applicable in the future.

The discovery of *B. lamudiae* demonstrates that limestone-associated habitats in northeastern Thailand remain underexplored for seasonal and localized Zingiberaceae. Continued field surveys, especially during the flowering season, are necessary for documenting narrowly distributed taxa, clarifying species limits, and supporting conservation planning. In this context, the description of *B. lamudiae* contributes not only to the taxonomy of *Boesenbergia*, but also to the broader documentation of plant diversity in limestone landscapes of Thailand.

### 4.5. Limitations and Future Perspectives

The present study is based on comparative morphology, repeated observations of living material, herbarium and literature comparisons, and phenetic analyses. Molecular phylogenetic data were not generated; therefore, the recognition of *B. lamudiae* should be regarded as a morphology-based taxonomic hypothesis rather than direct evidence of evolutionary independence. This limitation is important in *Boesenbergia*, particularly because *B. rotunda* sensu lato has been broadly circumscribed and shows considerable morphological variation across its range. Nevertheless, *B. lamudiae* is separated from the selected comparable taxa by a stable combination of independent characters, including the arachnoid abaxial lamina surface, maroon lanceolate bracts, long pubescent calyx with an acute to acuminate apex, non-saccate obovate labellum with a sharply delimited transverse colour zone, larger anther crest, longer epigynous glands, and pubescent ovary. Future molecular phylogenetic studies with broader sampling of *B. lamudiae*, *B. burmanica*, *B. rotunda* sensu lato, *B. baimaii*, *B. isanensis*, and other related taxa lacking an androecial cup will be necessary to test the phylogenetic placement and evolutionary independence of this species.

## 5. Conclusions

This study describes *Boesenbergia lamudiae* as a new limestone-associated species from Loei Province, northeastern Thailand. The species is clearly distinguished from morphologically similar taxa, especially *B. burmanica* and the selected operational units of *B. rotunda* sensu lato, by a unique combination of characters, including abaxially arachnoid laminae, maroon lanceolate bracts, longer pubescent calyx with an acute to acuminate apex, non-saccate obovate labellum with a sharply delimited transverse colour zone, larger anther crest, longer epigynous glands, and pubescent ovary. The phenetic analyses based on 25 selected morphological characters further supported its morphological distinctiveness, with *B. lamudiae* separated from all compared operational units in the PCoA and UPGMA analyses.

The discovery of *B. lamudiae* highlights the importance of detailed field observations, living material, and microhabitat-based surveys in the taxonomy of *Boesenbergia*. Several diagnostic floral characters, particularly labellum colour pattern, indumentum, anther crest morphology, and floral form, are difficult to interpret from dried material alone. The occurrence of this species in a limestone-associated habitat also emphasizes the potential of underexplored limestone landscapes in northeastern Thailand to harbour localized and previously overlooked plant diversity.

Although fewer than 50 mature individuals were observed at the only known locality, adjacent limestone areas remain insufficiently surveyed. Therefore, *B. lamudiae* is provisionally assessed as Critically Endangered (CR) under criterion D, pending additional field surveys to clarify its full distribution, population size, habitat specificity, and potential threats. Further exploration of limestone habitats in Loei and nearby provinces is likely to improve understanding of species diversity, endemism, and conservation priorities within Thai *Boesenbergia*. This taxonomic recognition is based on morphological and phenetic evidence, and molecular phylogenetic data will be needed in future studies to test the phylogenetic placement and evolutionary independence of *B. lamudiae*.

## Figures and Tables

**Table 1:** Diagnostic morphological comparison of *Boesenbergia lamudiae*, *B. burmanica*, and *B. rotunda* sensu lato.

Character	*B. lamudiae*	*B. burmanica*	*B. rotunda* Sensu Lato ^1^
Lamina	30–42 cm long 8–15 cm wide Broadly elliptic to ovate-elliptic, sometimes slightly oblong-elliptic	10–34 cm long 4–7.5 cm wide Narrowly elliptic	6–50 cm long 1.5–16.5 cm wide Variable; ovate-oblong, elliptic-lanceolate, elliptic or oblong
Flowers per spike	15–20	9–10	8–12
Bracts	Narrowly lanceolate to lanceolate Maroon	Oblong Pale green	Lanceolate to oblong Green, white, or translucent
Bracteoles	Lanceolate, 2.0–3.8 × 0.4–0.8 cm Translucent maroon	Oblong to lanceolate 4–4.3 × 0.6–0.8 cm Translucent greenish white	Elliptic, lanceolate, oblong-lanceolate 3–5.5 × 0.4–0.7 cm Translucent greenish white
Flower length	10.5–12 cm	8–9 cm	6–10 cm
Calyx length	3.2–3.6 cm	2.2–2.5 cm	0.7–2.0 cm
Calyx apex	Acute to acuminate	Bifid	Dentate, 2-cleft, tridentate, truncate, or bilobed
Floral tube	7.8–8.5 cm long	5.5–5.8 cm long	4–7.5 cm long
Dorsal corolla lobe	Lanceolate, 2.8–3.0 × 0.6–0.7 cm	Lanceolate, 2.2–2.4 × 0.7–0.8 cm	Oblong to lanceolate, 1.5–2.3 × 0.5–0.7 cm
Lateral corolla lobes	Lanceolate, 2.5–2.6 × 0.50–0.52 cm	Lanceolate, 2.1–2.2 × 0.6–0.7 cm	Oblong to lanceolate, ca. 1.5–2.0 × 0.4–0.5 cm
Lateral staminodes	Obovate 2.2–2.5 × 1.2–1.4 cm White Glabrous	Obovate 1.4–1.5 × 0.8–0.9 cm White Sparsely puberulous	Obovate to spathulate 1.4–2.0 × 0.5–1.3 cm Pale pink to pink Glabrous
Labellum shape	Obovate, not saccate	Oblanceolate, not saccate	Variable; shortly spathulate, fiddle-shaped, elliptic, ovate-elliptic, or obovate; slightly saccate, or saccate
Labellum size	3.2–3.5 × 2.0–2.2 cm	2.8–3.4 × 1.8–1.9 cm	1.5–3.5 × 0.8–1.7 cm
Labellum colour pattern	Basal portion white to creamy white, with a very pale pinkish tint along the central basal region and midline; throat with a sharply delimited deep reddish to maroon transverse band; distal portion pink to pale pink, darker toward the centre and apex, with conspicuously darker veins, rarely with small reddish spots	White with bright red towards apex and reddish spots in midlobe	Variable; white, pink, red, or violet, with diffuse, spotted, striped, or radiating markings
Anther crest	ca. 3 × 2.5 mm	ca. 1 × 1 mm	1–3 × 2 mm
Epigynous glands	10–12 mm long	7.4–8 mm long	5–8 mm long
Ovary	5–7 × 2.5–3 mm Pubescent	ca. 3 × 2 mm Glabrous	3–7 × 1.5–2 mm Glabrous or nearly glabrous
Habitat	Shaded limestone-associated habitat, sandy loam soil mixed with limestone rocks, 420–450 m a.s.l.	Sandy loam soil mixed with rocks in a shady area, at an elevation of approximately 140–145 m a.s.l.	Widespread; mixed deciduous or evergreen forests, limestone hills, along streams, and widely cultivated
Distribution	Known only from Loei Province, northeastern Thailand	Known only from Naypyidaw Union Territory, Myanmar	Widely distributed and cultivated across tropical Asia
References	Present study	[11]	[2,4,9,17,20,21,28,39]

^1^ Data for *Boesenbergia rotunda* sensu lato were compiled from published descriptions from Thailand, China, Myanmar, India, and Cambodia, including the protologues of taxa previously described as *B. baimaii* and *B. isanensis*. Following the treatment in the Flora of Thailand [17], material and descriptions published under these names are considered here as part of the morphological variation in *B. rotunda* sensu lato. This treatment is used only for comparative purposes and does not imply any taxonomic revision of these names in the present study.

## Data Availability

The morphological character definitions and coded character-state matrix used in the phenetic analyses are provided in Table A1 and Table A2. Additional data are available from the corresponding author upon reasonable request.

## Abbreviations

The following abbreviations are used in this manuscript:

A	Harvard University, Cambridge, Massachusetts, U.S.A.
AAU	Aarhus University, Denmark
B	Botanic Garden and Botanical Museum Berlin-Dahlem, Germany
BK	Bangkok Herbarium, Department of Agriculture, Thailand
BKF	Department of National Parks, Wildlife and Plant Conservation, Thailand
BM	The Natural History Museum, London, England, U.K.
C	University of Copenhagen, Copenhagen, Denmark
CAL	Botanical Survey of India, Howrah, West Bengal, India
DMSC	Medicinal Plants Research Institute, Thailand
E	Royal Botanic Garden Edinburgh, Edinburgh, United Kingdom
HITBC	Xishuangbanna Tropical Botanical Garden, Academia Sinica, Yunnan, China
HN	Vietnam Academy of Science and Technology (VAST), Hanoi, Vietnam
HNPM	National Institute of Medicinal Materials, Vietnam
HNU	VNU University of Science, Hanoi, Vietnam
IBK	Guangxi Institute of Botany, China
IMDY	Chinese Academy of Medical Sciences, China
K	Royal Botanic Gardens, United Kingdom
KKU	Khon Kaen University Herbarium, Thailand
L	Naturalis Biodiversity Center, Leiden, Netherlands
NY	The New York Botanical Garden, New York, U.S.A.
P	Muséum National d’Histoire Naturelle, Paris, France
QBG	Queen Sirikit Botanic Garden, The Botanical Garden Organization, Thailand
SGN	Southern Institute of Ecology, Ho Chi Minh City, Vietnam
SING	Singapore Botanic Gardens, National Parks Board, Singapore
TNS	National Museum of Nature and Science, Tokyo, Japan
VMSU	Vascular Plant Herbarium, Mahasarakham University, Thailand
VNM	Institute of Life Science, Ho Chi Minh City, Vietnam
VNMN	Vietnam National Museum of Nature, Hanoi, Vietnam

## Appendix A

**Table A1:** Morphological characters and character states used in the phenetic similarity analysis of selected *Boesenbergia* taxa lacking an androecial cup.

Code	Character ^1^	Character States
C1	Leaf sheath indumentum	0 = glabrous or glabrescent; 1 = puberulous, pubescent, villous, or hairy
C2	Petiole indumentum	0 = glabrous or glabrescent; 1 = puberulous, pubescent, villous, or hairy
C3	Lamina shape	0 = narrowly elliptic or elliptic-lanceolate; 1 = elliptic, oblong, or ovate-oblong; 2 = broadly elliptic to ovate-elliptic
C4	Lamina width	0 = mostly <8 cm wide; 1 = mostly 8–15 cm wide; 2 = mostly >15 cm wide
C5	Adaxial lamina indumentum	0 = glabrous or glabrescent; 1 = puberulous, pubescent, villous, or hairy
C6	Abaxial lamina indumentum	0 = glabrous or glabrescent; 1 = sparsely hairy, finely pubescent, pubescent, or villous; 2 = arachnoid
C7	Number of flowers per spike	0 = fewer than 8 flowers; 1 = 8–12 flowers; 2 = more than 12 flowers
C8	Bract shape	0 = oblong or elliptic; 1 = lanceolate to narrowly lanceolate
C9	Bract colour	0 = green, pale green, white, or translucent; 1 = reddish, maroon, or russet
C10	Bract indumentum	0 = glabrous or glabrescent; 1 = sparsely hairy, puberulous, puberulent, or pubescent
C11	Bracteole shape	0 = elliptic, oblong, or oblong-lanceolate; 1 = lanceolate
C12	Flower length	0 = <8 cm long; 1 = 8–10 cm long; 2 = >10 cm long
C13	Calyx length	0 = <2.0 cm long; 1 = 2.0–3.0 cm long; 2 = >3.0 cm long
C14	Calyx indumentum	0 = glabrous or glabrescent; 1 = sparsely hairy, puberulent, pubescent, or villous
C15	Calyx apex	0 = truncate, dentate, irregularly cleft, tridentate; 1 = bifid or bilobed; 2 = acute to acuminate
C16	Floral tube length	0 = ≤6.0 cm long; 1 = >6.0–7.5 cm long; 2 = >7.5 cm long
C17	Lateral staminode shape	0 = spathulate, oblong, or narrowly obovate; 1 = obovate or obovate-oblong; 2 = broadly obovate or orbicular
C18	Lateral staminode length	0 = ≤2.0 cm long; 1 = >2.0 cm long
C19	Lateral staminode colour	0 = white, creamy white, ivory, or very pale yellowish white; 1 = yellow, pale yellow, or yellow-orange; 2 = pink, pale pink, rose, purplish pink, or purple
C20	Labellum shape	0 = spathulate, elliptic, ovate, ovate-elliptic, or fiddle-shaped; 1 = oblanceolate; 2 = obovate; 3 = orbicular or nearly orbicular
C21	Labellum saccation	0 = saccate, slightly saccate, or slightly concave; 1 = not saccate
C22	Labellum colour pattern	0 = weakly differentiated, diffuse, or variable colour markings, without a clearly localized apical/midlobe colour concentration or a sharply delimited transverse colour boundary; 1 = spotted, striped, or radiating markings; 2 = red, pink, reddish, maroon, violet, or purplish colour mainly concentrated toward the apex, apical half, or midlobe, often with spots or darker patches, but without a sharply delimited transverse colour boundary; 3 = sharply delimited transverse colour band or colour zone separating differently coloured portions of the labellum
C23	Anther crest length	0 = ≤1.5 mm long; 1 = >1.5–2.5 mm long; 2 = >2.5 mm long
C24	Epigynous gland length	0 = ≤8 mm long; 1 = >8–<10 mm long; 2 = ≥10 mm long
C25	Ovary indumentum	0 = glabrous or nearly glabrous; 1 = puberulent, pubescent, or hairy

^1^ Characters were selected from vegetative and floral features consistently available from living material, protologues, published descriptions, regional floristic treatments, photographs, and illustrations. The presence or absence of an androecial cup was used as a criterion for selecting the operational units and was therefore not included as a coded character. Habitat, distribution, leafy shoot height, pseudostem height, and number of leaves per leafy shoot were excluded from the final matrix to reduce ecological, geographical, and ontogenetic bias. Colour characters were coded based on the dominant ground colour or the most clearly described or illustrated colour pattern in the cited source.

**Table A2:** Character-state matrix used for the phenetic similarity analysis of selected *Boesenbergia* taxa lacking an androecial cup.

Species ^1^	C1	C2	C3	C4	C5	C6	C7	C8	C9	C10	C11	C12	C13	C14	C15	C16	C17	C18	C19	C20	C21	C22	C23	C24	C25	Sources
*B. lamudiae*	1	1	2	1	0	2	2	1	1	1	1	2	2	1	2	2	1	1	0	2	1	3	2	2	1	Present study
*B. burmanica*	1	1	0	0	1	1	1	0	0	1	1	1	1	1	1	0	1	0	0	1	1	2	0	0	0	[11]
*B. meghalayensis*	1	1	1	1	0	0	0	0	0	0	1	1	1	0	0	1	1	0	0	0	0	2	1	1	0	[2]
*B. rotunda*—Thailand	0	0	1	1	0	1	1	0	0	1	0	0	0	0	0	0	1	0	2	0	0	2	0	0	0	[39]
*B. rotunda*—China ^2^	0	0	1	0	0	1	1	1	0	0	0	0	0	0	0	0	0	0	2	0	0	2	0	0	0	[4]
*B. rotunda*—Myanmar ^2^	0	0	1	1	0	1	1	1	0	0	0	0	0	0	0	0	0	0	2	0	0	2	0	0	0	[28]
*B. rotunda*—India	1	1	1	1	0	1	1	0	0	1	0	1	0	0	0	1	1	0	2	0	0	2	1	0	0	[2]
*B. rotunda*—Cambodia	0	0	1	1	0	1	1	0	0	1	0	1	0	0	0	1	1	0	2	0	0	2	1	0	0	[9]
*B. baimaii*	0	0	1	0	0	0	1	1	0	1	1	1	0	1	0	0	1	0	0	2	1	2	1	0	0	[21]
*B. isanensis*	1	1	1	0	0	1	0	1	0	1	1	0	0	1	0	0	0	0	0	2	1	3	0	0	0	[20]

^1^ The synonymy of *Boesenbergia rotunda* follows current taxonomic treatments; however, only selected published descriptions and protologues with sufficient comparable morphological information were coded as operational units. Other heterotypic synonyms of *B. rotunda* were not coded separately because the present analysis was designed as a focused phenetic comparison for species delimitation of *B. lamudiae*, not as a revision of *B. rotunda* synonymy. The inclusion of *B. baimaii* and *B. isanensis* is for comparative purposes only and does not imply taxonomic recognition or revision of these names in the present study. Character codes C1–C25 correspond to the morphological characters and character states defined in Table A1. ^2^ In some taxa, the lateral staminodes are described as white, whereas the accompanying illustrations show them as pale pink or pink, sometimes with a white basal portion and pale pink distal portion. For comparative purposes, the colour state was coded as pale pink or pink when this condition was clearly evident in the published illustrations.

## References

[1] POWO Plants of the World Online, Facilitated by the Royal Botanic Gardens, Kew. http://www.plantsoftheworldonline.org/.

[2] Aishwarya K., Vinitha M.R., Thomas G., Sabu M. (2015). A new species of *Boesenbergia* and rediscovery of *B. rotunda* (Zingiberaceae) from India. Phytotaxa.

[3] Larsen K., Ibrahim H., Khaw S.H., Saw L.G. (1999). Gingers of Peninsular Malaysia and Singapore.

[4] Chen J., Xia N.-H. (2019). A taxonomic revision of Chinese *Boesenbergia* (Zingiberaceae), with a new record. Phytotaxa.

[5] Mood J.D., Trần H.Đ., Veldkamp J.-F., Prince L.M. (2016). *Boesenbergia siphonantha* (Zingiberaceae)—A new record for Thailand and Vietnam with notes on molecular phylogeny. Gard. Bull. Singap..

[6] Yongco J.E., Amparado O.A., Naive M.A.K. (2023). Rediscovery of the Zamboanga Peninsula endemic *Boesenbergia longipetiolata* (Ridl.) Merr. (Zingiberaceae) after over a century, including an amended description and an updated distribution. Biodiversitas J. Biol. Divers..

[7] Saensouk P., Saensouk S., Boonma T., Oo W.P., Htet N.M., Maknoi C., Bongcheewin B., Htway N.N., Minn H.M. (2025). Two new records of *Boesenbergia* Kuntze (Zingibereae: Zingiberaceae) for the flora of Myanmar. Biodiversitas J. Biol. Divers..

[8] Tad-o R.V.B., Alafag J.I., Napaldet J.T. (2022). *Boesenbergia igorota* (Zingiberaceae), a new species from the Cordillera Central Range, northern Philippines. Taiwania.

[9] Saensouk P., Saensouk S., Boonma T., Song D., Maknoi C., Setyawan A.D. (2025). *Boesenbergia* Kuntze (Zingiberaceae) in Cambodia: Four new records with notes on their potential horticultural significance, cultivation guidelines, and lectotypification of *B. xiphostachya* (Gagnep.) Loes. Horticulturae.

[10] Larsen K. (1993). *Boesenbergia tenuispicata* (Zingiberaceae): A new species from Thailand. Nord. J. Bot..

[11] Saensouk P., Saensouk S., Boonma T., Htway N.N., Oo W.P., Naing M.K., Junsongduang A. (2025). A New Species of *Boesenbergia* Kuntze (Zingiberaceae) from Myanmar, with Notes on Diversity, Utilization, Conservation, and Horticultural Potential. Taxonomy.

[12] Boonma T., Saensouk S. (2019). *Curcuma saraburiensis* (Zingiberaceae), a new species from Thailand. Taiwania.

[13] Boonma T., Saensouk S., Saensouk P. (2020). Two new species of *Kaempferia* L. (Zingiberaceae) from Thailand. Taiwania.

[14] Boonma T., Saensouk S., Saensouk P. (2024). Biogeography, conservation status, and traditional uses of Zingiberaceae in Saraburi Province, Thailand, with *Kaempferia chaveerachiae* sp. nov.. Horticulturae.

[15] Triboun P., Keeratikiet K. (2016). *Zingiber sirindhorniae*, a remarkable new species in *Zingiber* section *Dymczewiczia* (Zingiberaceae) from Thailand. Thail. Nat. Hist. Mus. J..

[16] Mood J.D., Veldkamp J.-F., Dey S., Prince L.M. (2014). Nomenclatural changes in Zingiberaceae: *Caulokaempferia* is a superfluous name for *Monolophus* and *Jirawongsea* is reduced to *Boesenbergia*. Gard. Bull. Singap..

[17] Mood J.D., Veldkamp J.-F., Newman M.F., Barfod A.S., Esser H.J., Simpson D., Parnell J.A.N. (2023). Boesenbergia. Flora of Thailand.

[18] Chinese Virtual Herbarium (CVH) Institute of Botany, Chinese Academy of Sciences. http://www.cvh.ac.cn.

[19] (2026). Index Herbariorum. A Worldwide Index of Herbaria and Associated Staff Where Plant and Fungal Specimens are Permanently Housed.

[20] Saensouk S., Saensouk P. (2020). *Boesenbergia isanensis* (Zingiberaceae), a new species from Thailand. Jpn. J. Bot..

[21] Saensouk S., Larsen K. (2001). *Boesenbergia baimaii*, a new species of Zingiberaceae from Thailand. Nord. J. Bot..

[22] Sabu M., Prasanthkumar M.G., Škorničková J., Jayasree S. (2004). Transfer of *Kaempferia siphonantha* Baker to *Boesenbergia* Kuntze (Zingiberaceae). Rheedea.

[23] Mood J.D., Prince L.M., Veldkamp J.-F., Dey S. (2013). The history and identity of *Boesenbergia longiflora* (Zingiberaceae) and descriptions of five related new taxa. Gard. Bull. Singap..

[24] Meekiong K., Lim C.K. (2014). The unusual branching *Boesenbergia* from Belum, Perak, and notes on the genus in Peninsular Malaysia. Folia Malays..

[25] Mood J.D., Veldkamp J.-F., Prince L.M. (2014). A new species and a new record of *Boesenbergia* (Zingiberaceae) for Thailand. Gard. Bull. Singap..

[26] Izzaty M.M., Tawan C.S., Meekiong K. (2015). *Boesenbergia atropurpurea*, a new *Boesenbergia* species from Sarawak, Malaysia. Folia Malays..

[27] Aishwarya K., Sabu M. (2015). *Boesenbergia pulcherrima* and *B. tiliifolia* (Zingiberaceae) in India: Notes on the identity, variability and typification. Rheedea.

[28] Mood J.D., Tanaka N., Aung M.M., Murata J. (2016). The genus *Boesenbergia* (Zingiberaceae) in Myanmar with two new records. Gard. Bull. Singap..

[29] Lý N.-S. (2017). *Boesenbergia quangngaiensis* (Zingiberaceae), a new species from Central Vietnam. Phytotaxa.

[30] Mood J.D., Trần H.Đ., Veldkamp J.-F., Prince L.M. (2018). Taxonomy of *Boesenbergia parvula* (Zingiberaceae) with new synonymy. Thai For. Bull. Bot..

[31] Mood J.D., Veldkamp J.-F., Mandáková T., Prince L.M., de Boer H.J. (2019). Three new species of *Boesenbergia* (Zingiberaceae) from Thailand and Lao P.D.R. Gard. Bull. Singap..

[32] Mood J.D., Ardiyani M., Veldkamp J.-F., Mandáková T., Prince L.M., de Boer H.J. (2020). Nomenclature changes in Zingiberaceae: *Haplochorema* is reduced to *Boesenbergia*. Gard. Bull. Singap..

[33] Docot R.V.A., Santiago L.C.P., Funakoshi H., Lam N.F. (2020). Two new species of *Boesenbergia* (Zingiberaceae) from Palawan, Philippines. Edinb. J. Bot..

[34] Aishwarya K., Sabu M. (2021). On the IUCN status of *Boesenbergia albolutea* and *B. rubrolutea* (Zingiberaceae) and typification of *B. rubrolutea*. J. Threat. Taxa.

[35] Lam N.F., Ibrahim H., Sam Y.Y., Zakaria R.M., Poulsen A.D. (2022). Two new species of *Boesenbergia* (Zingiberaceae) from Sabah, Malaysia. PhytoKeys.

[36] Tanaka N., Paing C.S., Aung M.M. (2024). Taxonomic studies of Zingiberaceae in Myanmar V: A new species of *Boesenbergia* from Rakhine State. Bull. Natl. Mus. Nat. Sci. Ser. B Bot..

[37] Santharam S.T., Kaliamoorthy S. (2024). *Boesenbergia kalakadensis* sp. nov. (Zingiberaceae) from Southern Western Ghats, India. Nord. J. Bot..

[38] Debnath S., Patil S., Vijayan D. (2024). *Boesenbergia ashihoi* (Zingiberaceae), a new species from northeast India. Nord. J. Bot..

[39] Sirirugsa P. (1992). A revision of the genus *Boesenbergia* Kuntze (Zingiberaceae) in Thailand. Nat. Hist. Bull. Siam Soc..

[40] Beentje H. (2016). The Kew Plant Glossary, an Illustrated Dictionary of Plant Terms.

[41] International Plant Names Index. https://www.ipni.org/.

[42] Sneath P.H.A., Sokal R.R. (1973). Numerical Taxonomy.

[43] Gower J.C. (1971). A general coefficient of similarity and some of its properties. Biometrics.

[44] Hammer Ø., Harper D.A.T., Ryan P.D. (2001). PAST: Paleontological Statistics Software Package for Education and Data Analysis. Palaeontol. Electron..

[45] IUCN (2024). Guidelines for Using the IUCN Red List Categories and Criteria.

[46] Kaufman L., Rousseeuw P.J. (1990). Finding Groups in Data: An Introduction to Cluster Analysis.

[47] Clements R., Sodhi N.S., Schilthuizen M., Ng P.K.L. (2006). Limestone karsts of Southeast Asia: Imperiled arks of biodiversity. BioScience.

[48] Kiew R., Rahman R.A. (2021). Plant diversity assessment of karst limestone, a case study of Malaysia’s Batu Caves. Nat. Conserv..

[49] Tan J.P.C., Kiew R., Darbyshire I. (2024). Prioritising Important Plant Areas (IPAs) among the limestone karsts of Perak, Malaysia. Kew Bull..

[50] Tiwari H., Arya D., Sekar K.C. (2025). Population status, threats, and conservation of *Trachycarpus takil*: An endemic and threatened plant species in western Himalaya, India. J. Threat. Taxa.

